# Transcription factor 3 promotes migration and invasion potential and maintains cancer stemness by activating ID1 expression in esophageal squamous cell carcinoma

**DOI:** 10.1080/15384047.2023.2246206

**Published:** 2023-08-21

**Authors:** Zhao-Xing Li, Ming-Chuang Sun, Kang Fang, Zi-Ying Zhao, Zhu-Yun Leng, Ze-Hua Zhang, Ai-Ping Xu, Yuan Chu, Li Zhang, Jingjing Lian, Tao Chen, Mei-Dong Xu

**Affiliations:** a Endoscopy Center, Department of Gastroenterology, Shanghai East Hospital, School of Medicine, Tongji University, Shanghai, China; b Department of Pathology, Shanghai East Hospital, School of Medicine, Tongji University, Shanghai, China

**Keywords:** TCF3, ID1, cancer stem cell, esophageal squamous cell carcinoma, cancer stemness

## Abstract

Transcription factor 3 (TCF3) is a member of the basic Helix – Loop – Helix (bHLH) transcription factor (TF) family and is encoded by the TCF3 gene (also known as E2A). It has been shown that TCF3 functions as a key transcription factor in the pathogenesis of several human cancers and plays an important role in stem cell maintenance and carcinogenesis. However, the effect of TCF3 in the progression of esophageal squamous cell carcinoma (ESCC) is poorly known. In our study, TCF3 was found to express highly and correlated with cancer stage and prognosis. TCF3 was shown to promote ESCC invasion, migration, and drug resistance both from the results of in vivo and in vitro assays. Moreover, further studies suggested that TCF3 played these roles through transcriptionally regulating Inhibitor of DNA binding 1(ID1). Notably, we also found that TCF3 or ID1 was associated with ESCC stemness. Furthermore, TCF3 was correlated with the expression of cancer stemness markers CD44 and CD133. Therefore, maintaining cancer stemness might be the underlying mechanism that TCF3 transcriptionally regulated ID1 and further promoted ESCC progression and drug resistance.

## Introduction

Esophageal squamous cell carcinoma (ESCC) is an aggressive cancer and its incidence has been increasing in these years, especially in China. The poor prognostic outcome is mainly caused by rapid cancer progression. Many patients have been identified with late cancer stage and metastasis at the time of diagnosis. Metastasis limits the chances for curative endoscopic submucosal dissection (ESD)^1^, which is a minimally invasive and standard treatment for early-stage alimentary tract tumors. Therefore, it is necessary to clarify the mechanism underlying the cancer progression, as well as find potential molecular markers to predict advanced ESCCs.

Cancer metastasis depends on a small subset of tumor cells (cancer stem cells) endowed with unlimited self-renewal, high invasive properties, and resistance to standard cancer therapies^2,3^. Transcription factor 3 (TCF3) belongs to the *T*-cell factor/lymphoid enhancer factor (TCF/LEF) transcription factor (TF) family and is encoded by the TCF3 gene (also known as E2A). TCF3 functions as a critical transcription factor in embryonic development, stem cell maintenance, and carcinogenesis^4^. Increasing evidence reveals that TCF3 plays an important role in the pathogenesis of several types of human cancers^4,5^. In the present study, we used the sequencing technique, combined with the TCGA database, and finally found that TCF3 was highly expressed in ESCC tissues. Furthermore, the overexpressed TCF3 level could predict advanced ESCC stages. In mechanism, elevated expression of TCF3 in cancer cells led to the upregulation of Inhibitor of DNA binding 1 (ID1) and finally promoted ESCC cell growth by inducing cancer stemness and enhancing the expression of CD44 and CD133. These results suggest that TCF3 promotes cancer progression by activating cancer stemness induced by ID1 in human ESCCs.

## Materials and methods

### Cell lines and culture conditions

The human ESCC cell lines including KYSE-150, KYSE-30, KYSE-410, TE-1, and ECA-109 were purchased from the Shanghai Institutes for Biological Sciences, Chinese Academy of Sciences and were routinely tested for mycoplasma contamination. Cells were cultured in DMEM medium (GIBCO) supplemented with 10% FBS (GIBCO) and 1% Penicillin-Streptomycin at 37°C in 5% CO_2_.

### Small interfering RNA (siRNA) and short hairpin RNA (shRNA) experiments

Corresponding TCF3 siRNA and ID1 siRNA were purchased from GenePharma, and Small Interfering RNA (siRNA) Experiments were performed as in our previous study^6^. Quantitative Real-time PCR and western blotting were performed after transfection. For shRNA experiments, lentivirus vectors that encode a shRNA targeting TCF3 were used to transfect KYSE150 cells following the manufacturer’s instruction (Genechem, Shanghai, China). The related sequences were shown in TableS1.

### Cell invasion and migration assays

In migration assay, 5 × 10^5^ esophageal squamous cell carcinoma cells were resuspended in a 200 µl FBS-free medium and were added to the top chamber. As the chemical attractants, 500 µl complete medium was added to the lower chamber then. After incubation for 48 hours, we used 4% paraformaldehyde to fix the cells on the lower surface of the non-coated membrane and then stained them with crystal violet. After that, we used a light microscope (100×) to take three representative field images from each membrane. The cells were counted according to standard procedures. In invasion assay, the only difference was that a gel made from Matrigel matrix (Corning, 356234) according to the manufacturer’s instruction was added in the top chamber before cells were plated.

### Cell counting kit-8 (CCK-8) proliferation assay

In CCK8 assay, 2 × 10^4^ cells were plated per well of 96 wells plate. After 0, 24, 48, 72, and 96 hours of incubation, 10 µl of CCK-8 reagent was added per well and the absorbance at 450 nM was analyzed by a microplate reader.

### Cisplatin incorporation assay

In cisplatin incorporation assay, 2 × 10^4^ cells were plated in the per well of 96 wells plate. After cells were adhered to the plate, different concentrations of cisplatin were added and cells were incubated for 24 hours. Cell absorbance analysis was the same as the CCK-8 experiment.

### Wound healing migration assay

A scratch assay was performed when ESCC cells grew to about 80% confluency. The scratch experiment was performed in 6-well plates by the tip of a 200 μl pipette. Images were collected at the same location at 0 h and 24 h respectively after scratch. The images were analyzed and the closure was calculated by ImageJ. The scratch experiments were performed in triplicate.

### RNA extraction, real-time PCR assay, and RNA sequencing

Total RNA was isolated using TRIzol reagent. TCF3 and ID1 were subjected to PCR assays using reagents from Takara Bio. After the reactions were accomplished, a set threshold was used to define the CT values. Data were analyzed using the 2^−^^△^^△^^CT^ method. Each experiment has been repeated three times. RNA sequencing was carried out by Genechem Biotechnology (Shanghai) Co., Ltd. The related sequences were shown in Table S1.

### Chromatin immunoprecipitation assay

Upstate biotechnology kits were used to perform chromatin immunoprecipitation assays. Cells were treated according to the instruction provided by the kit manufacturer and quantitative real-time PCR was performed to measure the amount of bound DNA, and enrichment values were calculated based on the relative amount of input and the ratio to IgG. The related sequences were shown in Table S1.

### Dual luciferase reporter assay

ESCC cells were plated in a 12-well plate. The reporter plasmid containing promoters at the binding site were transfected into cells. Renilla luciferase reporter plasmids for signal normalization were also transfected. After incubation for 24 hours, the Dual Luciferase Assay System Kit (Promega, Madison, WI, USA) was used to measure the luciferase activity.

### Immunoblotting analysis

Proteins were collected from the cultured cells and then blotted by the antibodies mentioned below. We used SDS-PAGE to separate proteins, transferred them to PVDF membranes, and examined them with the relevant primary antibodies specific for TCF3 (ab229605), ID1 (ab168256), CD133 (ab278053), CD44 (ab189524), β-Actin(ab8227) was used as a loading control. Antibodies were purchased from Abcam.

### Immunofluorescence

Immunofluorescence was performed following the instruction of Abcam. Briefly, cells were permeabilized with 0.15% Triton X-100, incubated overnight at 4°C with the corresponding primary antibody, incubated for 30 minutes at room temperature in a dark environment with fluorescence secondary antibody, stained with DAPI reagent, and photographed under a fluorescent microscope, and analyzed by ImageJ.

### Immunohistochemistry

This study was approved by the Institutional Review Board of East Hospital, Tongji University (NO.2019072, Date of Approval:2019.09.06). Human tumor samples and their paired non-cancer paraneoplastic tissues were obtained from 79 patients with esophageal squamous cell carcinoma. Each patient agreed to participate in the relevant study and signed the informed consent. Survival time was calculated from the day of surgery to the date of death or the last follow-up visit. Tumor-node-metastasis (TNM) staging was confirmed based on the American Joint Committee on Cancer (AJCC) criteria. Results were scored by two pathologists, and the clinical data were kept confidential from them. The analysis was performed as before^7,8^.

### Flow cytometric

CD44+/CD44- cells were sorted from KYSE-150 cells. FACS was performed with > 1 × 10^6^ cells using the Beckman CytoFLEX. All flow cytometric analyses were performed using built-in software (Beckman CytoFLEX). CD44 antibody purchased from R&DSystems (FAB6127G).

### Animals

KYSE-150 cells infected by anti-TCF3-LV or anti-NC-LV were harvested, suspended (with serum-free medium), and injected into the right mid-posterior axilla of each mouse (5 × 10^6^ tumor cells/mouse). The mice were divided into two different groups (*n* = 5 each). After 5 weeks, the mice were sacrificed and the subcutaneous tumors were excised and removed. The tumor volume and weight were measured by an electronic scale and vernier caliper. One portion of the tumor tissue was used for protein extraction and the other portion was fixed in 10% paraformaldehyde and paraffin-embedded for IHC assay. The Animal Care and Use Committee of Tongji University approved the relevant animal experiments^6^.

## Results

### TCF3 is highly expressed in esophageal squamous cell carcinoma

**Figure 1.**
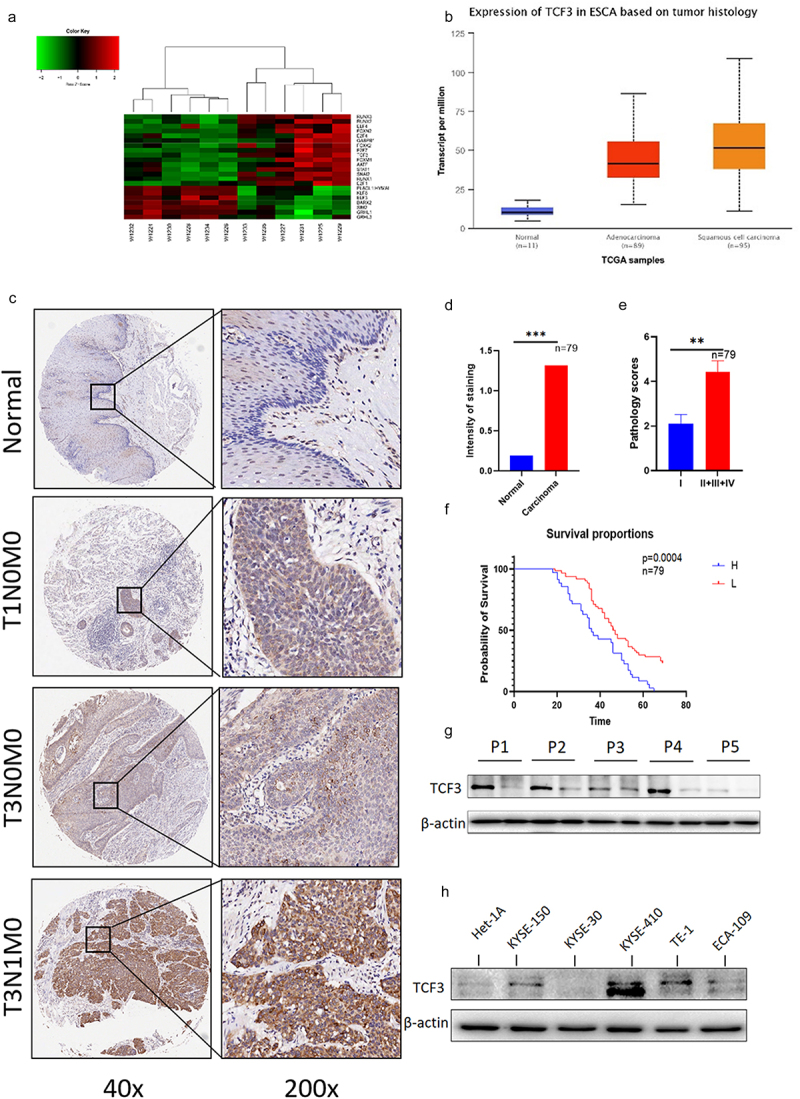
TCF3 is highly expressed in esophageal squamous cell carcinoma and correlates with patient prognosis. a. 6 pairs of esophageal squamous cell carcinoma tissue microarray shows that TCF3 is highly expressed in esophageal carcinoma compared to normal tissues and the expression increases with increasing malignancy. b. TCGA database shows that TCF3 expression in ESCC is highly expressed compared to other genes. c. Esophageal squamous cell carcinoma tissue microarray from 79 patients shows that TCF3 is highly expressed in ESCC tissues compared to normal tissues. d-e. The expression level of TCF3 is corresponding to ESCC progression. f. High TCF3 expression correlates with the prognosis of ESCC.g.TCF3 protein is highly expressed in ESCC tissues of five randomly selected patients. h. TCF3 is higher in ESCC cell lines especially in KYSE-150, TE1, and KYSE-410 than in normal esophageal epithelial cell line HET-1A.

TCF3 is highly expressed in the hematologic system^9,10^, but in solid tumors, especially ESCC, the expression of TCF3 is still obscure. We sequenced the tumors and adjacent tissues of six pairs of patients using sequencing technology. Then we screened all highly expressed transcription factors (Figure 1A). We wanted to explore the relationship between transcription factors and stemness, so we tested the related genes using TCGA database and found that TCF3 had higher expression in esophageal squamous cell carcinoma (Figure 1B). We performed IHC of TCF3 in collected carcinoma and paraneoplastic tissues and found that TCF3 was significantly upregulated in ESCC tissues and mostly expressed in nucleus. Moreover, we also found that the expression of TCF3 was correlated with the progression and prognosis of ESCC (Figure 1C–F). Additionally, the expression level of TCF3 protein in ESCC tissues was further confirmed by immunoblotting analysis in five randomly selected ESCC patients (Figure 1G). Also by Immunoblotting analysis, it was obvious that the expression level of TCF3 was higher in ESCC cell lines, especially in KYSE-150, TE1, and KYSE-410 than in normal esophageal epithelial cell line HET-1A (Figure 1H).

*p < .05, **p < .01. ***p < .001. NC, negative control.

### TCF3 promotes the migration, proliferation, and invasion of ESCC cells

**Figure 2.**
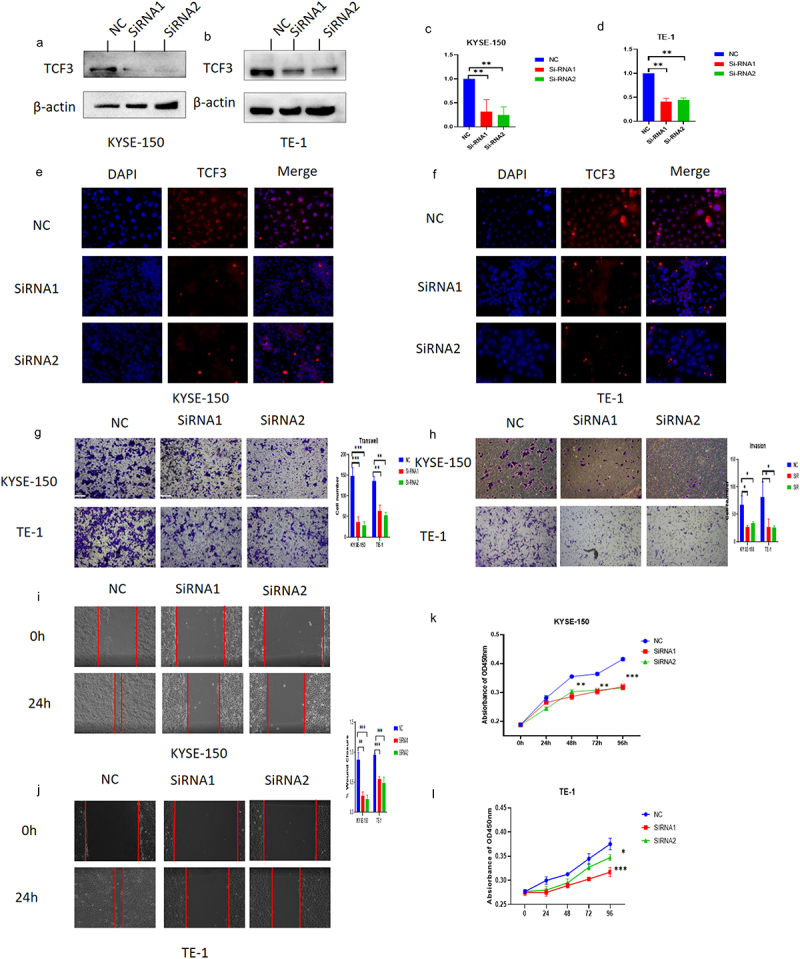
TCF3 promotes proliferation, migration, and invasion of ESCC cells. a-d. KYSE-150 and TE-1 have decreased TCF3 protein expression after siRnas targeting TCF3 could significantly knockdown the expression of TCF3 protein and TCF3 mRNA. E-F. The fluorescence intensity of TCF3 was reduced in KYSE-150 and TE-1 after si-TCF3 was transfected. G-H. The migration and invasion abilities of KYSE-150 and TE-1 were decreased when TCF3 was knocked down. I&J. The wound healing rates of KYSE-150 and TE-1 were decreased when TCF3 was knocked down. K&L. Cell proliferation is affected by KYSE-150 and TE-1 after the siRNA knockdown of TCF3.

We knocked down TCF3 by Si-RNA in selected ESCC cell lines TE-1 and KYSE-150 and verified the knockdown efficiency by RT-PCR, immunoblotting analysis, and immunofluorescence (Figure 2A–F). Meanwhile, the results of immunofluorescence further confirmed that TCF3 was mostly expressed in nucleus ESCC cells which matched the IHC results of TCF3 in collected carcinoma and paraneoplastic tissues (Figure 2E,F). Subsequently, we explored the effect of TCF3 on the ESCC cells, as was shown in the transwell assay and the wound healing assay, we found that TCF3 could promote the migration and invasion ability of ESCC cells (Figure 2G–J). In the CCK8 assay, the proliferation ability of esophageal squamous cell carcinoma cells was reduced after TCF3 knockdown (Figure 2K,L).

*p < .05, **p < .01. ***p < .001. KNC, KYSE-150 negative control. KS, KYSE-150 siRNA knockdown. TNC, TE-1 negative control. TS, TE-1 siRNA knockdown.

### TCF3 affects the stemness of esophageal squamous cell carcinoma by regulating ID1

**Figure 3.**
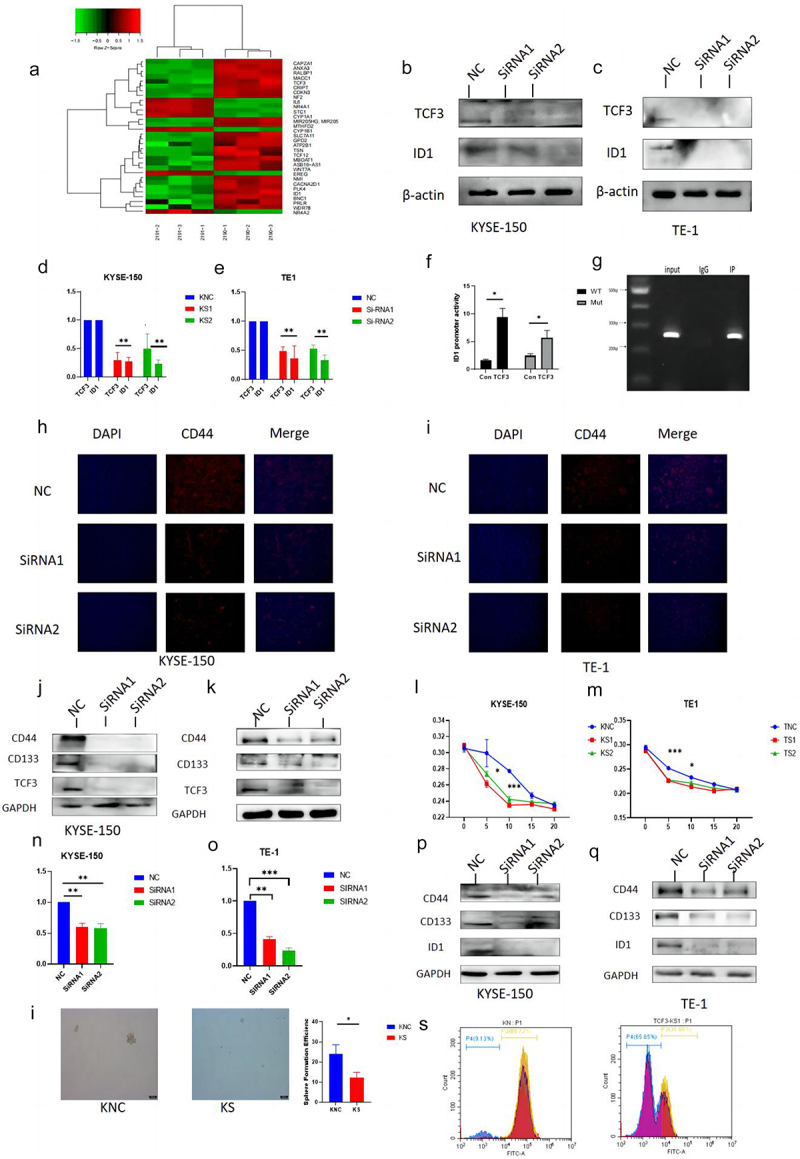
TCF3 regulates the expression of cancer stem markers CD44 and CD133 by transcriptionally regulating ID1. a. RNA-seq results show significantly upregulated and downregulated gene signatures. b-c. In KYSE-150 and TE-1, with the knockdown of TCF3 the protein expression level of ID1 is subsequently reduced. d-e. In KYSE-150 and TE-1, with the knockdown of TCF3, the mRNA expression of ID1 is subsequently reduced. f-g. CHIP and Dual luciferase reporter assay suggested TCF3 could transcriptionally regulate ID1.h-i. With knockdown of TCF3 in KYSE-150 and TE-1 CD44, fluorescence intensity decreased. J-K. The protein expression of CD44 and CD133 was reduced with the knockdown of TCF3 in KYSE-150 and TE-1. l -m. With the knockdown of TCF3 in KYSE-150 and TE-1 knockdown increased sensitivity to the chemotherapeutic drug cisplatin. n-o. mRNA expression of ID1 was reduced in KYSE-150 and TE-1 after siRNA knockdown. p-q. The protein expression level of CD44 and CD133 was subsequently reduced in KYSE-150 and TE-1 when ID1 was knocked down. I. KYSE-150 has a significant decrease in the sphere formation efficiency after si-TCF3 was transfected. s. with knockdown of TCF3, CD44+ ESCC cells number were decreased.

We performed an RNA-seq assay on ESCC cells after the knockdown of TCF3 and found that it may regulate ID1 after relevant measurements (Figure 3A), so we determined the mRNA and protein expression levels of ID1 after TCF3 was knocked down, and the results suggested that the expression level of both ID1 protein and mRNA decreased subsequently with the knockdown of TCF3 in TE-1 and KYSE-150 cell lines (Figure 3B–E). To explore the regulatory relationship between TCF3 and ID1, we performed Lucifers and Chip experiments, which demonstrated that TCF3 was bound to the promoter of gene ID1 and directly regulates the expression of ID1 (Figure 3F,G). The analysis of the results of RNA-seq revealed that TCF3 may regulate the process of tumor development through the regulation of tumor stemness. Moreover, it has been suggested that ID1 was associated with cancer stemness^11,12^. While CD44 and CD133 have been recognized as stemness markers for esophageal squamous cell carcinoma cells^13,14^. We measured the effect of TCF3 on the expression of CD44 and CD133 by western-blot assay and immunofluorescence assay. We found that the fluorescence signal of CD44 was diminished (Figure 3H,I), and their protein expression decreased with the knockdown of TCF3 (Figure 3J,K), which suggested that TCF3 was indeed associated with stemness markers of ESCC. The determination of its resistance by cisplatin stimulation showed that more cells died at lower concentrations in the knockdown group than in the control group, suggesting that the knockdown group was more sensitive to the chemotherapeutic drug cisplatin (Figure 3L,M). Therefore, TCF3 may be related to drug resistance in ESCC. Meanwhile, to explore the effect of ID1 on the stemness of ESCC, we knocked down the expression of ID1 in ESCC cell lines KYSE-150 and TE-1 (Figure 3N,O). We found that the expression of CD44 and CD133 was also reduced when ID1 was knocked down (Figure 3P,Q). Meanwhile, KYSE-150 has a significant decrease in the sphere formation efficiency after si-TCF3 was transfected (Figure 3I), and the cell flow cytometry assay showed that the number of CD44+ cells decreased after knockdown of TCF3 (Figure 3S). To further investigate whether TCF3 promotes ESCC progression by regulating ID1, we performed rescue assays. We overexpressed ID1 in TCF3-knockdown ESCC cells (Fig S1, A&amp;B), and found that overexpression of ID1 could help restore the relevant function, which was decreased by knockdown of TCF3 (FigS1, C-I). The results above suggested that TCF3 affected the stemness of esophageal squamous cell carcinoma by regulating ID1.

*p < .05, **p < .01. ***p < .001. KNC, KYSE-150 negative control. KS, KYSE-150 siRNA knockdown. TNC, TE-1 negative control. TS, TE-1 siRNA knockdown.

### ID1 promotes the migration, proliferation, and invasion of ESCC

**Figure 4.**
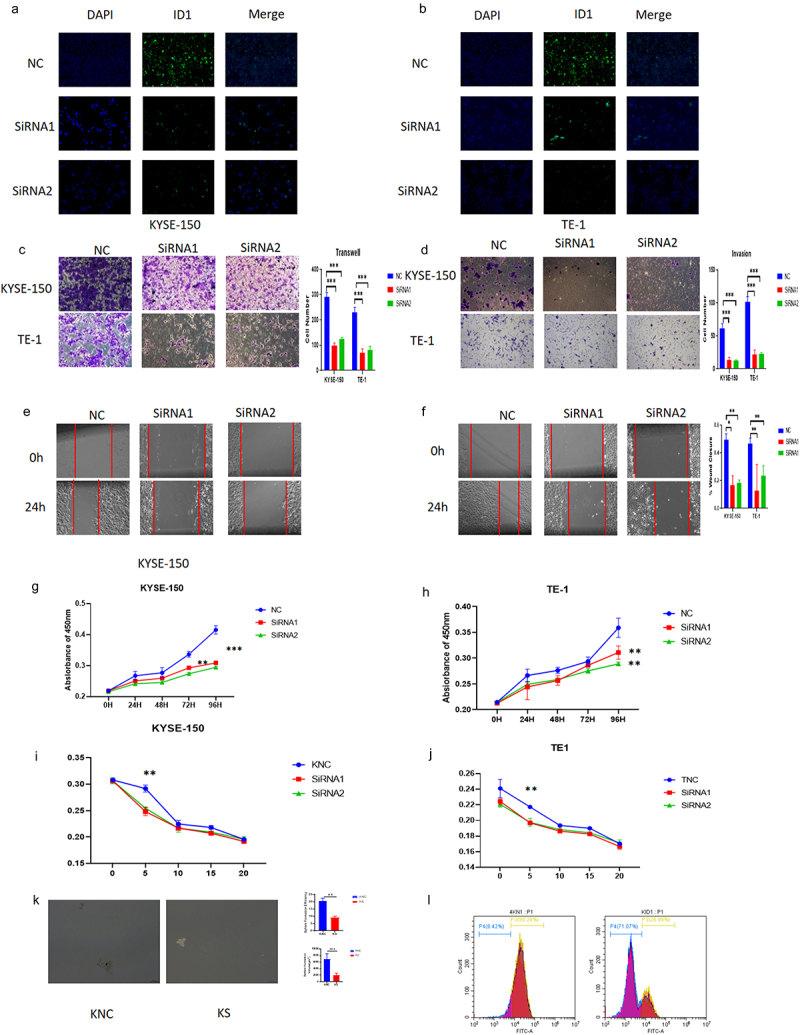
ID1 promotes proliferation, migration, invasion, and drug resistance of ESCC cells. a-b. The fluorescence intensity of ID1 was reduced in KYSE-150 and TE-1 after ID1 was knocked down. c-d. The migration and invasion abilities of KYSE-150 and TE-1 were decreased when ID1 was knocked down. e -f. The wound healing rates of KYSE-150 and TE-1 were decreased when ID1 was knocked down. g-h. Cell proliferation is affected in KYSE-150 and TE-1 after the siRNA knockdown of ID1.I&J. Increased sensitivity to the chemotherapeutic drug cisplatin with knockdown of TCF3 in KYSE-150 and TE-1. k. KYSE150 has a significant decrease in the sphere formation efficiency after si-ID1 was transfected. l. with knockdown of ID1, CD44+ ESCC cells number were decreased.

After ID1 was knocked down by siRNA, its fluorescence intensity was subsequently diminished (Figure 4A,B). We then investigated the effect of ID1 on the development of ESCC cells. From the results of the transwell assay and wound healing assay, we found that ID1 could significantly promote the migration and invasion of ESCC cells (Figure 4C–F). After the CCK8 assay was performed, we found that ID1 could augment the proliferation ability of ESCC cells (Figure 4G,H). The determination of the drug resistance by cisplatin stimulation showed that the knockdown group was more sensitive to the chemotherapeutic drug cisplatin (Figure 4I,J), which suggested that ID1 might help ESCC resist common chemotherapeutic therapy clinically. Tumor sphere assay shows sphere formation efficiency has highly decreased after si-ID1 was transfected (Figure 4K). Cell flow cytometry assay shows CD44+ ESCC cells have a significant decrease after ID1 was knockdown (Figure 4L). These results suggest that ID1 affects the stemness of ESCC again.

*p < .05, **p < .01. ***p < .001. KNC, KYSE-150 negative control. KS, KYSE-150 siRNA knockdown.TNC, TE-1 negative control.TS, TE-1 siRNA knockdown.

### Tumor stemness markers express highly in ESCC

**Figure 5.**
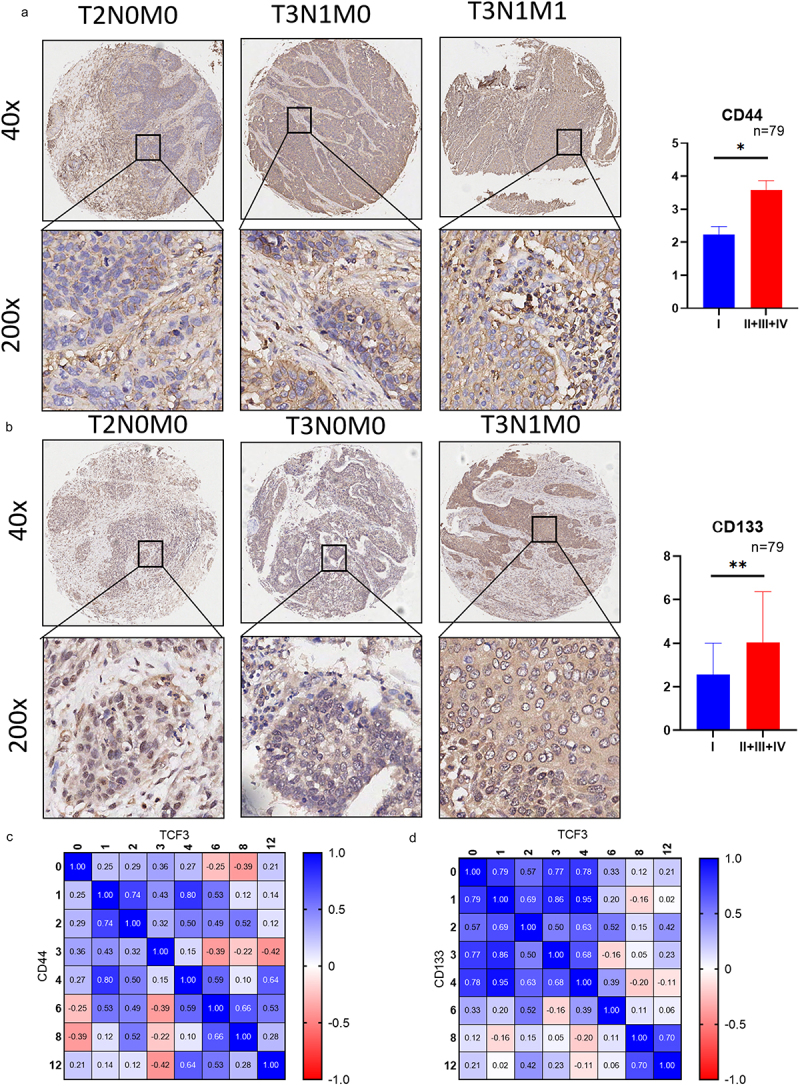
The expressions of cancer stem markers CD44 and CD133 were correlated with the progression of ESCC. a. The expression of CD44 is corresponding to the tumor staging of ESCC. b. The expression of CD133 is corresponding to the tumor staging of ESCC. c. The expression of CD44 positively correlates with TCF3 expression.d.CD133 positively correlates with the expression of TCF3. *p < .05, **p < .01. ***p < .001.

We then determined the expression of tumor stemness markers CD44 and CD133 in ESCC by immunohistochemistry. The results showed that CD44 and CD133 were highly expressed in ESCC tissues and the expression level was also correlated with tumor staging. (Figure 5A,B). In addition, the correlation analysis between TCF3 and CD44 or CD133 showed that the expression of CD44 and CD133 was correlated with TCF3 (Figure 5C,D).

### TCF3 affects the progression of ESCC in vivo

**Figure 6.**
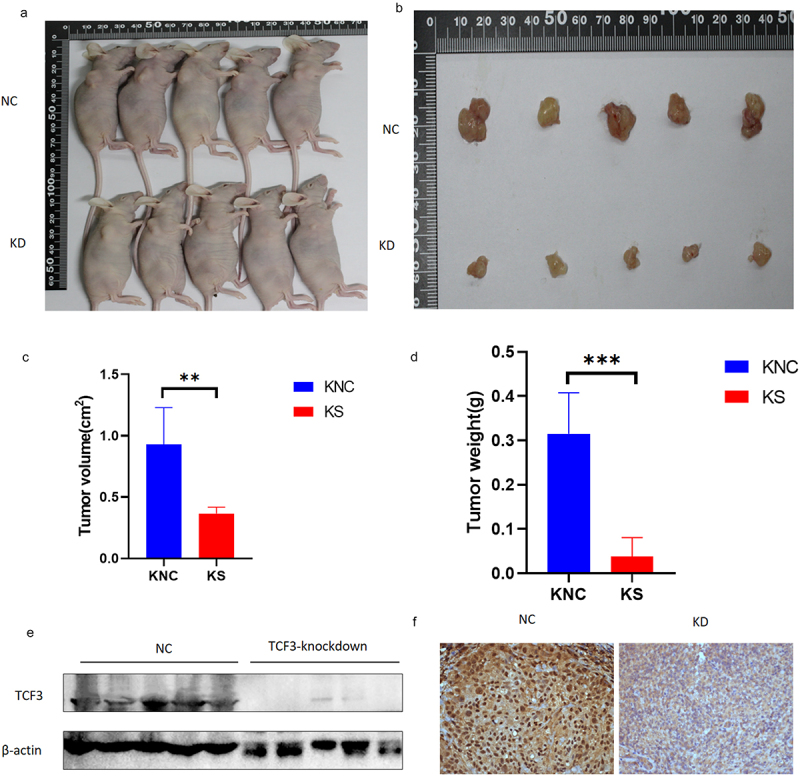
TCF3 promotes the progression of ESCC in vivo. a-b. Tumor appearance in mice after 1 month of subcutaneous tumor formation. c. Smaller tumor volume in the knockdown group compared to the NC group. D. Smaller tumor weight in the knockdown group compared to the NC group. e-f. Reduced TCF3 expression in the knockdown group compared to the NC group.

We then explored the effects of TCF3 in vivo. We constructed TCF3-knockdown KYSE-150 cell lines and control cell lines by stable transfection and injected the same number of cells into the right mid-posterior axilla of each mouse. After we injected ESCC cells in mice, we checked the status of the mice and measured the size of tumors once a day, and after 4 weeks of culturation, mice were sacrificed and tumor tissue was removed. We measured the size and weight of the tumors and found that the tumors were smaller in size and weight in the TCF3-knockdown group than those in the control group (Figure 6A–D). We also determined the expression of TCF3 in the tumor tissues by immunoblotting analysis and immunohistochemistry, the results of which confirmed that TCF3 does promote the progression of ESCC in vivo (Figure 5E,F).

*p < .05, **p < .01. ***p < .001. KNC, KYSE-150 negative control. KS, KYSE-150 siRNA knockdown. TNC, TE-1 negative control. TS, TE-1 siRNA knockdown.

## Discussion

Esophageal cancer has a high morbidity in China, and is often accompanied by high aggressiveness and risk of recurrence^15^. In previous studies, TCF3 has been shown to be associated with tumor development in many other cancer species^16,17^, however, little is known about the role of TCF3 in ESCC. We demonstrated that TCF3 was indeed associated with invasive metastasis and the prognosis of ESCC. TCF3 was more highly expressed in ESCC compared with paraneoplastic tissues. In addition, as the stages of ESCC increased, the expression of TCF3 also increased. Moreover, TCF3 was demonstrated to be associated with survival time in patients. In ESCC cells, downregulated TCF3 by siRNA inhibited the migration, proliferation, and invasive functions of KYSE-150 and TE-1. Finally, our study indicated that TCF3 has an important function in the development of ESCC. Our data demonstrated that TCF3 might be a predicting factor for the prognosis and development of ESCC.

Inhibit or of DNA binding 1 (ID1) is a helix-loop-helix (HLH) protein that forms heterodimers with the members of the basic HLH family of TFs. Proteins of HLH family contain an HLH dimerization domain which is composed of two conserved amphipathic α helices separated by a loop and an adjacent region that contacts DNA^18^. Basic HLH (bHLH) proteins are bound to a DNA sequence known as an E-box or to the related N-box, which is found in the promoter-enhancer of expressed genes^19^. Of all the ID proteins, ID1 is extensively studied and mostly linked to cancer malignant behavior and poorer prognosis^20,21^. In this study, by RNA-Seq, we found the regulatory relationship between TCF3 and ID1, which was verified by Luciferase and Chip. We also verified the role of ID1 in the development of ESCC by using siRNA knockdown. Our data suggested that ID1 has an effect on the migration, proliferation, and invasive function of ESCC cells. In addition, we constructed the TCF3 knockdown strain of KYSE-150 by virus and again verified the regulatory role of TCF3 in animal experiments.

Esophageal cancer is a highly lethal malignancy, and although many traditional treatment modalities are available, new treatment modalities still need to be explored. Cancer stemness is thought to be related to cancer progression, drug resistance, and resistance to radiation therapy. The problems that how to modulate the stemness of cancer cells and further reduce drug and radiation therapy resistance of cancer cells need to be urgently investigated. It has been suggested that the anti-chemotherapy function of cancer cells can be regulated by adjusting the stemness of cancer cells^22^. Through our study, we demonstrated that TCF3 modulated the stemness of ESCC by regulating ID1, and also demonstrated that the sensitivity of KYSE-150 and TE-1 to the chemotherapeutic drug cisplatin, which is one of the most commonly used chemotherapeutic drugs for treating esophageal cancer, was increased after knockdown of TCF3 or ID1 by siRNA. Therefore, our experiments can provide new targets and research ideas for the study of chemoresistance in ESCC. We demonstrated that inhibition of TCF3 greatly abolished tumor sphere-forming capacity in vitro and tumorigenicity in vivo induced by the TCF3-ID1 axis. Meanwhile, targeting the TCF3-ID1 axis could be an effective means to combat ESCC.

## Conclusions

In conclusion, our study indicates that TCF3 has an important function in the development of ESCC and reveals that TCF3 regulates the cancer progression by activating cancer stemness induced by ID1.

## Supplementary Material

Supplemental Material

Supplemental Material

Supplemental Material

## Data Availability

The data that support the findings of this study are available from the corresponding author, Dr Li & Dr Xu, upon reasonable request.
