# Supplementary material for: Transcription factor 3 promotes migration and invasion potential and maintains cancer stemness by activating ID1 expression in esophageal squamous cell carcinoma
Source: Cancer Biol Ther. 2023 Aug 21;24(1):2246206. doi: 10.1080/15384047.2023.2246206 (PMC10443991; doi:10.1080/15384047.2023.2246206)
Supplement: Supplemental Material [file KCBT_A_2246206_SM0708.docx]

FigS1. Overexpression ID1 in ESCC cells which TCF3 were knockdown could help to restore migration and invasion potential functions. A-B. Overexpression ID1 in ESCC cells which TCF3 was knockdown has no effect on the expression of TCF3. C. Overexpression ID1 could help to restore migration functions. D-E. The wound healing rates of KYSE-150 and TE-1 were restored when ID1 was overexpressed. F-G. Overexpression ID1 could help to restore cell proliferation function. H-I. Overexpression ID1 could help to improve sensitivity to the chemotherapeutic drug cisplatin

*p<0.05, **p<0.01. ***p<0.001. KNC, KYSE-150 negative control. KS, KYSE-150 siRNA knockdown. TNC, TE-1 negative control.TS, TE-1 siRNA knockdown. OE, overexpression
