# Supplementary figures and images for: Transcription factor 3 promotes migration and invasion potential and maintains cancer stemness by activating ID1 expression in esophageal squamous cell carcinoma
Source: Cancer Biol Ther. 2023 Aug 21;24(1):2246206. doi: 10.1080/15384047.2023.2246206 (PMC10443991; doi:10.1080/15384047.2023.2246206)

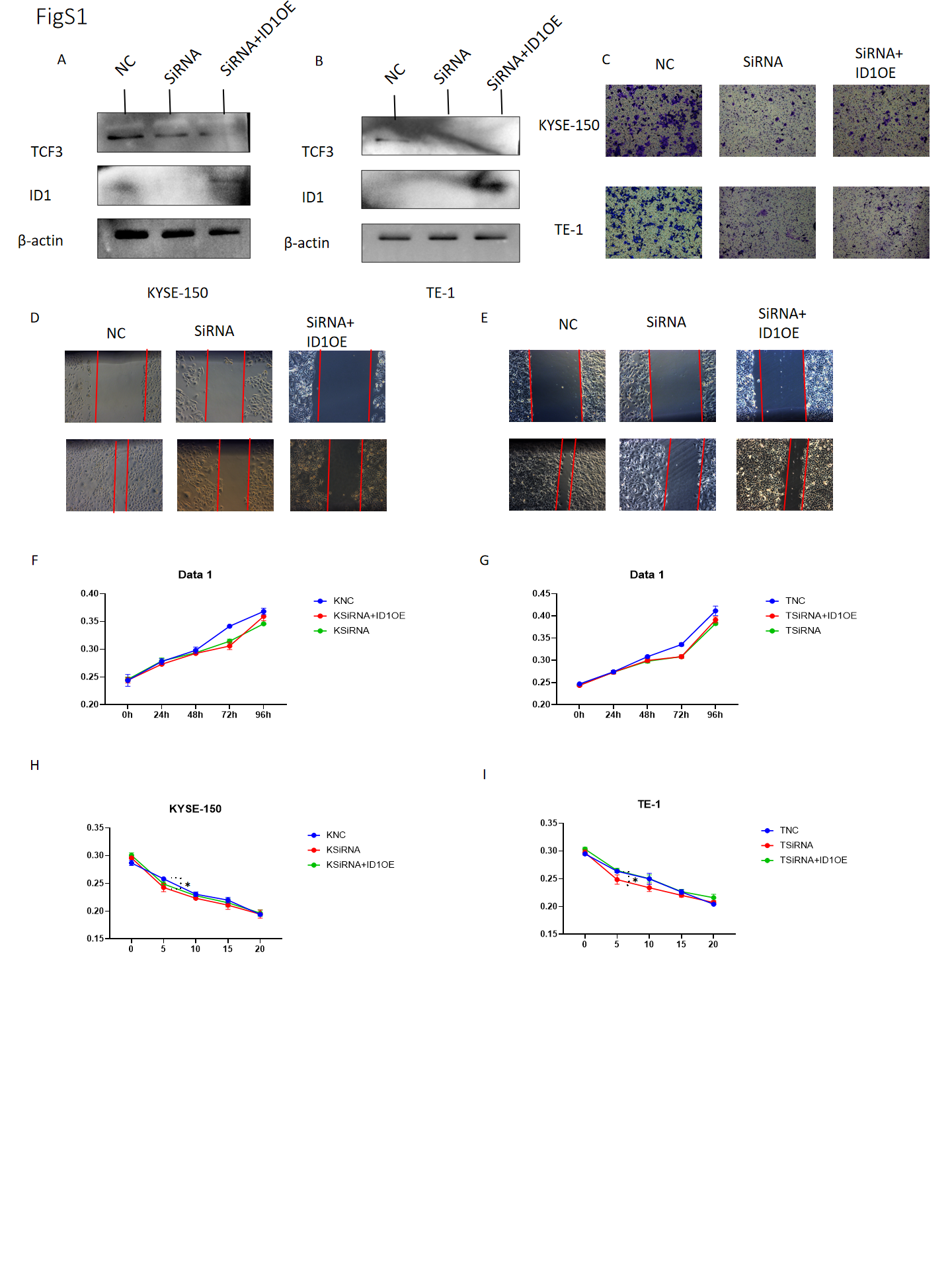

Supplement: Supplemental Material [file KCBT_A_2246206_SM0707.tif]
