# Supplementary material for: Transcription factor 3 promotes migration and invasion potential and maintains cancer stemness by activating ID1 expression in esophageal squamous cell carcinoma
Source: Cancer Biol Ther. 2023 Aug 21;24(1):2246206. doi: 10.1080/15384047.2023.2246206 (PMC10443991; doi:10.1080/15384047.2023.2246206)
Supplement: Supplemental Material [file KCBT_A_2246206_SM0706.docx]

| assay | name | Sequence |
| --- | --- | --- |
| siRNA | NC-sense | 5’-UUCUCCGAACGUGUCACGUTT-3’ |
|  | NC-antisense | 5’-ACGUGACACGUUCGGAGAATT-3’; |
|  | TCF3-siRNA1 | GCCUCUCUUCAUCCACAUUTTAAUGUGGAUGAAGAGAGGCTT |
|  | TCF3-siRNA2 | CAAUAACUUCUCGUCCAGTTCUGGACGAGAAGUUAUUGCTT |
|  | ID1-siRNA1 | GGGAUUCCACUCGUGUGUUTTAACACACGAGUGGAAUCCCTT |
|  | ID1-siRNA2 | UGGACGAGCAGCAGGUAAATTUUUACCUGCUGCUCGUCCATT |
| real-time PCR | TCF3-F | GCACCCTCCCTGACCTGTCTC |
|  | TCF3-R | GTTCTCCTCGTCCTCCTTCTCCTC |
|  | ID1-F | CTACGACATGAACGGCTGTTA |
|  | ID1-R | CAACTGAAGGTCCCTGATGTAG |
| Chromatin immunoprecipitation | ID1-F | CTGCTTAGCTTCCTTGCCTCC |
|  | ID1-R | TTATAAACACGCCACGGCCC |
| Overpression | ID1-p1 | CACACTGGACTAGTGGATCCCGCCACCATGAAAGTCGCCAGTGGCAG |
|  | ID1-p2 | AGTCACTTAAGCTTGGTACGCGACACAAGATGCGATCGTCCG |

Table S1 .Sequence of each assay
